# Characterization of a Subunit of the Outer Dynein Arm Docking Complex Necessary for Correct Flagellar Assembly in *Leishmania donovani*

**DOI:** 10.1371/journal.pntd.0000586

**Published:** 2010-01-26

**Authors:** Simone Harder, Meike Thiel, Joachim Clos, Iris Bruchhaus

**Affiliations:** Bernhard Nocht Institute for Tropical Medicine, Hamburg, Germany; Yale School of Public Health, United States of America

## Abstract

**Background:**

In order to proceed through their life cycle, *Leishmania* parasites switch between sandflies and mammals. The flagellated promastigote cells transmitted by the insect vector are phagocytized by macrophages within the mammalian host and convert into the amastigote stage, which possesses a rudimentary flagellum only. During an earlier proteomic study of the stage differentiation of the parasite we identified a component of the outer dynein arm docking complex, a structure of the flagellar axoneme. The 70 kDa subunit of the outer dynein arm docking complex consists of three subunits altogether and is essential for the assembly of the outer dynein arm onto the doublet microtubule of the flagella. According to the nomenclature of the well-studied *Chlamydomonas reinhardtii* complex we named the *Leishmania* protein *Ld*DC2.

**Methodology/Principal Findings:**

This study features a characterization of the protein over the life cycle of the parasite. It is synthesized exclusively in the promastigote stage and localizes to the flagellum. Gene replacement mutants of *lddc2* show reduced growth rates and diminished flagellar length. Additionally, the normally spindle-shaped promastigote parasites reveal a more spherical cell shape giving them an amastigote-like appearance. The mutants lose their motility and wiggle in place. Ultrastructural analyses reveal that the outer dynein arm is missing. Furthermore, expression of the amastigote-specific A2 gene family was detected in the deletion mutants in the absence of a stage conversion stimulus. *In vitro* infectivity is slightly increased in the mutant cell line compared to wild-type *Leishmania donovani* parasites.

**Conclusions/Significance:**

Our results indicate that the correct assembly of the flagellum has a great influence on the investigated characteristics of *Leishmania* parasites. The lack of a single flagellar protein causes an aberrant morphology, impaired growth and altered infectiousness of the parasite.

## Author Summary

*Leishmania* parasites are responsible for the disease leishmaniasis. They are spread through sandflies. The primary hosts are mammals, including humans. They occur in two different morphological forms. The flagellated promastigotes live in the gut of the sandfly vector. After transmission to the mammalian host they get phagocytized by macrophages and convert into the amastigote form, which is able to survive within the phagolysosome. The molecular mechanisms underlying this transformation process from promastigote to amastigote are poorly understood so far. A striking difference of the life cycle stages is a long flagellum in the promastigote compared to only a rudimentary flagellum in the mammalian stage amastigote. During an earlier study of the stage differentiation of *Leishmania donovani* we identified a flagellar protein, a subunit of the outer dynein arm docking complex (ODA-DC2). This protein is part of a flagellar structure called the axoneme. Here we have further characterized the protein regarding its role within the life cycle of the parasite. Mutant promastigotes lacking DC2 protein show reduced flagellar length and a more amastigote-like appearance overall. In addition, the motility is heavily retrenched and transmission electron microscopy indicated that the flagellar ultrastructure is affected. Furthermore, the mutants express amastigote-specific genes and show increased *in vitro* infectiousness towards macrophages. Therefore, we conclude that the correct assembly of the flagellum is vital for maintenance of the promastigote stage of the parasite.

## Introduction

Protozoan parasites of the genus *Leishmania* cause a variety of diseases in humans collectively termed as leishmaniasis. The pathologies range from self-healing cutaneous lesions (*Leishmania major*) to fatal visceral involvement (*Leishmania donovani*). Two million new infections are estimated to occur annually, with an estimated 12 million people presently infected in over 85 endemic countries worldwide [1]. The parasite is transmitted to mammalian hosts as the infective flagellated promastigote form from the gut of its insect vector, female phlebotomine flies. Promastigotes are phagocytized by macrophages wherein they develop into tamastigote form which is able to survive and proliferate inside the fully acidified phagolysosomes of their host cells [2]. The developmental stage differentiation is mainly induced by changes in pH and temperature and each stage is highly adapted for extra- or intracellular survival in the specific environment encountered in insect and vertebrate host [3]. One aspect of the transformation from promastigote to amastigote parasites is the regulation of organelle and overall cell size. The promastigotes are spindle-shaped cells with a long flagellum protruding from the flagellar pocket, an invagination of the cytoplasmic membrane at the anterior end of the cell. By contrast, the amastigotes display a more spherical form with an overall reduced cellular volume and only a rudimentary flagellum that does not protrude from the flagellar pocket. The flagellum is involved in various processes such as cell motility but also attachment to host surfaces and intracellular signaling [4],[5].

As in most motile eukaryotic flagella, a canonical “9+2” microtubule axoneme drives the flagellar movement of *Leishmania* parasites. It consists of nine outer doublet microtubules (A- and B-tubule) surrounding a pair of centrally located singlet microtubules. Radial spokes extend inward from each outer doublet towards the central pair. ATP-dependent dynein motor proteins attached to each doublet translocate along the adjacent doublet to generate the sliding force that underlies flagellar movement. Cilia and flagella of eukaryotic cells contain three different classes of dyneins: cytoplasmic ones as well as the inner and outer dynein arms of the axoneme. *L. mexicana* contains two cytoplasmic dynein-2 heavy chain genes (*LmxDHC2.1+2.2*) and a single dynein-1 heavy chain gene *(LmxDHC1)*. Disruption of *LmxDHC2.2* results in an amastigote-like phenotype and immotility of the parasite. Nevertheless, protein expression is still as in the promastigote stage. Further studies indicate the absence of the paraflagellar rod proteins PFR1 and PFR2 and that the *LmxDHC2.2* is required for correct flagellar assembly [6].

Every dynein binds to a structurally unique binding site mediating a high specificity that is essential for the flagellar movement. The unicellular green algae *Chlamydomonas reinhardtii* serves as a model organism for studying the composition and function of flagella. Their outer dynein arms are very well characterized [7],[8]. These dyneins produce 80% of the flagellar force and bind to specific sites of the A-tubule of the outer microtubule doublet [9]. The globular heads possess a binding site for the B-tubule, and they are spread along the whole length of the axoneme with a regular distance of 24 nm. The outer dynein arms consist of several polypeptide chains: three heavy chains (HCα, β and γ), two intermediary chains (IC78 and IC69) and multiple light chains (LC1-8) [10]. In 1994, Takada and Kamiya could identify a protein complex responsible for the association of the outer dynein arm to the microtubule, the outer dynein arm docking complex (ODA-DC) [11]. Subsequent studies showed that this complex consists of three proteins present in equimolar amounts and in a 1∶1 stoichiometry with the outer dynein arm polypeptide chains [12]–[14]. The subunits DC1 [13] and DC2 [14] have coiled-coil domains and are wound around each other in an α-helical manner. The third subunit DC3, member of an EF hand superfamily of Ca^2+^ binding proteins, is also essential for the composition of the outer dynein arm and the ODA-DC [12].

The flagella of leishmania parasites reveal, apart from the above described axonemal structure, an additional peculiar characteristic feature: the paraflagellar rod (PFR). This is a unique network of cytoskeletal filaments which extends along the whole axonene within the flagella of kinetoplastids, euglenoids and dinoflagellates [15]. It was shown in *L. mexicana* and *T. brucei* that this structure is essential for the cellular movement [16],[17]. However, nothing is known about its function so far.

Here, we report the characterization of *Ld*DC2, a protein of the outer dynein arm docking complex (ODA-DC) from *Leishmania donovani*, a structure important for the integrity of the flagellar axoneme. The protein was identified during an earlier performed proteome analysis of *L. donovani* stage differentiation [18]. It is expressed exclusively during the promastigote stage of the parasite and localizes primarily to the flagellum. Deletion mutants display an altered morphology, impaired growth and show slightly increased *in vitro* infectivity.

## Materials and Methods

### Cultivation of cells

*L. donovani* 1SR strain, a gift from D. Zilberstein (Department of Biology, Technicon, Israel Institute of Technology, Haifa, Israel), was used for all experiments. Promastigotes (day 0) frozen directly after passage trough BALB/c mice were thawed and cultivated at 25°C in M199 medium supplemented with 25% fetal calf serum and 20 µg/mL gentamycin. *In vitro* differentiation to amastigotes was achieved as described previously [19]. Briefly, promastigotes (day 0) were heat-shocked at 37°C for 24 h (day 1) and then cultivated for up to 5 days at 37°C in mildly acidic medium (pH 5.5, day 2–5). Cell densities were determined using a CASY 1-Cell Counter & Analyser (Schaerfe Systems).

### PEC infection assay (intracellular amastigotes)

Peritoneal exudate cells (PECs) from 4–6 weeks old female C57black/6 mice were used for infection assays. Mice were treated with 5% thioglycolate in PBS given intraperitoneal four days prior to experiment. On day 4 mice were sacrificed and PECs were prepared by rinsing the peritoneum with 10 mL of sterile PBS. PECs were washed once and seeded at a density of 10^6^ cells per well in a 12-well plate on coverslips in RPMI-medium supplemented with 10% fetal calf serum, 5 mM glutamine and 50 µg/mL gentamycin. After incubation under 5% CO_2_ at 37°C for 24 hours, PECs were incubated with *L. donovani* parasites at a parasite to PEC ratio of 10∶1 for 48 hours. Non-engulfed parasites were washed away three times with warm RPMI and cells on coverslips were stained with Giemsa and used for microscopic studies. To assess infection rates, the quantities of overall PECs versus infected cells were determined. At least 400 cells in three independent experiments were assessed. All counts were done with coded samples to prevent bias.

Animal care and experimentation were performed in accordance with the German Federal Animal Protection Laws, in particular §§ 4, 7 and 10a, in the animal facility of the Bernhard Nocht Institute.

### Genomic DNA isolation

Genomic DNA from *L. donovani* logarithmic promastigotes was prepared using the Puregene DNA Purification System (Gentra Systems) according to the manufacturer's recommendations.

### Cloning and sequencing of *lddc2* gene

Two primers were designed based on the sequence of the *L. major* gene 5852119 (hypothetical protein CAB55364): sense primer CAB-S27 (5′-GAGACATATGTCAGTGGTGGCTGCCAA-3′); antisense primer CAB-AS27 (5′-GAGAGGATCCCTATTTGGCCTTCTGAG -3′). CAB-S27 and CAB-AS27 were used to PCR-amplify *L. donovani* genomic DNA (95°C for 1 min, 50°C for 1 min, 72°C for 2 min; 30 cycles using the Perkin Elmer DNA Thermal Cycler 480). The amplified product (1857 bp) was gel-purified and cloned into the pCR 2.1-TOPO vector. The gene was sequenced using the Big Dye Terminator PCR cycle sequencing kit as per the manufacturer's instructions (Applied Biosystems).

### RNA isolation and Northern blot analysis

RNA from *L. donovani* promastigotes and *in vitro* differentiated amastigote cells was isolated by subjecting the parasites to repeated cycles of freezing and thawing in TRIzol. For Northern blotting, agarose gels were loaded with 20 µg of total RNA. After transfer to a nylon membrane, the blots were sequentially hybridized with radio-labeled *lddc2* and *ß-tubulin* probes. Hybridizations were performed in 0.5 M Na_2_HPO_4_, 7% SDS, and 1 mM EDTA (pH 7.2) at 70°C. Blots were washed in 40 mM Na_2_HPO_4_ and 1% SDS (pH 7.2) at 70°C.

### Expression and purification of recombinant protein

The PCR-amplified DNA fragment coding for *lddc2* full-length protein was cloned into the prokaryotic expression plasmid pJC45, a derivative of pJC40 [20], using the restriction enzymes *Nde*I and *Bam*HI. Following transformation in *E. coli* BL21(DE3) [pAPlacIQ] the protein was expressed following standard procedures. Recombinant protein was isolated using Ni-NTA resin according to the manufacturer's recommendations (Qiagen, Hilden, Germany).

### Generation of polyclonal antibodies

200 µg of recombinant *Ld*DC2 was injected subcutaneously into a chicken. The first injection was done in combination with complete Freund's adjuvant, the following two booster injections were done in combination with incomplete Freund's adjuvant at two-week-intervals. Antibodies were purified from eggs using increasing concentrations of polyethyleneglycol 6000 [21].

### Western blot analysis

10% SDS-PAGE was performed under reducing conditions. Samples from promastigotes and *in vitro* derived amastigotes were obtained by lysing the cells directly in hot SDS sample buffer (95°C, 125 mM Tris-HCl pH 6.8, 20% glycerine, 20% SDS, 20 mM DTT, 0.001% bromophenolblue). Western Blot analyses were carried out using the semidry blotting technique with electrophoresis buffer (0.25 M Tris, 0.5 M glycine, 1% SDS) as blotting buffer. Polyclonal chicken antibodies (*Ld*DC2 1∶500) or monoclonal mouse antibodies (Anti-β tubulin clone Tub 2.1 (Sigma) and an alkaline phosphatase conjugated anti-chicken IgM or anti-mouse IgG (Sigma), as secondary antibody, were used to detect the protein with the 5-bromo-4-chloro-3-indolyl-phosphate (BCIP)/nitro blue tetrazolium (NBT) color developmental substrate (Promega).

### DNA constructs for homologous recombination

Primers CAB-5′UTR(S38)E/S (5′-GAGAATTCATTTAAATCCAAGCAAAGGCGAATACATAT-3′); CAB-5′UTR(AS37)B/K (5′-GAGGATCCGGTACCGACCAAGTCCACCAATGTACG-3′) and CAB-3′UTR(S31)B (5′-GAGGATCCGCGACAGCATGCCAGCAACACGG-3′) and CAB-3′UTR(AS37)H/S (5′-GAAAGCTTATTTAAATTCTGCGTAGCCTGTGTGTGG-3′) were used to PCR amplify the 5′UTR and 3′UTR of CAB55364 from genomic *L. major* DNA. The plasmid pUC19 was used as a cloning vector. *Δlddc2:neo* and *Δlddc2:pac* were constructed by ligating the 5′UTR-PCR-fragment into the *Eco*RI and *Bam*HI restriction sites followed by ligation of the 3′UTR-PCR-fragment into the *Bam*HI and *Hind*III restriction sites of the pUC19 vector. The selection markers neomycinphosphotransferase (*neo*) and puromycinacetyltransferase (*pac*) were ligated via integrated restriction sites for *Kpn*I (at the end of 5′UTR fragment) and *Bam*HI (at the beginning of 3′UTR fragment). Before transfection, the integration constructs were separated from the vector backbone by digestion with the enzyme *Swa*I.

### Construction of expression vectors

The *Leishmania*-specific expression vector pX63pol (kindly provided by Dr. Martin Wiese, Strathclyde Institute of Pharmacy and Biomedical Science, Glasgow, Scotland) was used to express *lddc2* in *L. donovani Δlddc2* ^n/p^ promastigotes. The two primers CAB-S27 and CAB-AS27 were used to PCR-amplify the coding region of *lddc2*. The product was digested with *Nde*I and *Bam*HI, the 5′overlapping ends were filled in by using Klenow polymerase to create blunt ends. The vector was digested with *Eco*RV and ligated with the prepared insert. The correct orientation and sequence was re-confirmed by nucleotide sequencing.

### Transfection of *L. donovani* promastigotes

Plasmid-DNA was purified by using the Nucleobond AX PC2000 Maxiprep-Kit (Macherey & Nagel). For episomal expression 100 µg of DNA was used per transfection; 5 µg of DNA was used for integration via homologous recombination. Parasites were transfected by means of electroporation. Cells were harvested during late log phase of growth, washed twice in ice-cold PBS, once in prechilled electroporation buffer (21 mM HEPES pH 7.5, 137 mM NaCl, 5 mM KCl, 0.7 mM Na_2_HPO_4_, 6 mM glucose) and suspended at a density of 1×10^8^ cells/mL in electroporation buffer. Chilled DNA was mixed with 0.4 mL of the cell suspension, which was immediately used for electroporation using a Bio-Rad Gene Pulser. Electrotransfection was carried out in a 4 mm electroporation cuvette at 3.750 V/cm and 25 microfarads. After electroporation, cells were kept on ice for 10 min before being transferred into 10 mL of antibiotic-free medium. After 24 h, the transfectants were selected with either 50 µg/mL G418 (neomycin), 25 µg/mL puromycin B or 7.5 µg/mL of bleomycin.

### Immunofluorescence assays (IFA)

*L. donovani* promastigotes were added to poly-(L-lysine) covered glass slides and air dried. Cells were fixed with 3.7% formaldehyde in M199 for 15 min, washed three times in PBS, and permeabilized in PBS/0.2% Triton-×100, and washed three three times in PBS. Subsequently, cells were incubated for 30 min in PBS containing 10% FCS. After blocking, cells were incubated with anti-*Ld*DC2 (1∶500), anti-β-tubulin (1∶500) or anti-PFR2 (1∶4) antibodies (provided by Martin Wiese), diluted in PBS/10% FCS, following three washes in PBS. Slides were incubated with Cy™2-conjugated anti-chicken IgG antibodies, Cy™2-conjugated anti-mouse IgG antibodies or Cy™3-conjugated anti-mouse IgG antibodies (Dianova), diluted 1∶1000 in PBS/10%FCS and washed another three times in PBS. After incubation with Hoechst 33258 (Molecular Probes, 1∶2000 in PBS), cells were mounted in mounting medium (Dako Cytomation) and examined with a Zeiss Axioskop 2 plus immunofluorescence microscope and using the OpenLab software package (Improvision).

### Electron and light microscopy

Scanning electron microscopy (SEM) was performed on *L. donovani* promastigotes that were harvested by centrifugation (10 min, 720×*g*, 4°C), washed twice with PBS and fixed with 2% glutaraldehyde in 0.1 M sodium cacodylate buffer (pH 7.2) and postfixed with 1% OsO_4_. Cells were dehydrated in increasing ethanol concentrations (30–100%), subjected to critical point drying, coated with gold, and viewed with a Philips SEM 500 electron microscope.

For transmission electron microscopy (TEM), cells were treated as described above and dehydrated with graded ethanol solutions and propylene oxide. Parasites were embedded in an epoxy resin (Epon). Ultrathin sections (70 nm) were cut (Ultra Cut E; Reichert/Leica, NuBlock, Germany) and counterstained with uranyl acetate and lead citrate. Sections were examined with a FEI TECNAI SPIRIT transmission electron microscope at an acceleration voltage of 80kV.

Phase contrast microscopy and flagellar length determination were performed on a Zeiss Axioskop 2 plus microscope. Parasites were stained with Giemsa and analyzed microscopically. The flagellar length was measured from the cell body to the tip of the flagellum using the ImageJ software.

## Results

### Cloning of the 70 kDa subunit of the outer dynein arm docking complex (ODA-DC) of *L. donovani* and analysis of its amino acid sequence

In the course of a proteome analysis of the *in vitro* stage differentiation of *L. donovani*, the hypothetical protein CAB55364 was found to be expressed in an amastigote-specific manner [18]. Primers deduced from the coding region of the orthologous *L. major* gene (accession no. 5852119) were used to amplify the corresponding DNA by PCR from *L. donovani* genomic DNA. The product obtained comprised 1857 bp and showed 96% sequence identity to its *L. major* homologous. Southern blot analysis indicated that the investigated gene is single-copy per haploid genome (data not shown). It encodes a hypothetical protein of 618 amino acid residues, a calculated *M_r_* of 70,000 and a pI value of 5.1. The protein is a putative homologous of the 70 kDa subunit of the outer dynein arm docking complex of *Chlamydomonas reinhardtii* (*Cr*DC2) [14]. This protein complex consists of three subunits and is essential for the assembly of the outer dynein arm onto the doublet microtubule of *C. reinhardtii* flagella. *C. reinhardtii*, an unicellular, biflagellate green algae of the order Volvocales, serves as a model organism for studying eukaryotic cilia and flagella.

**Figure 1:**
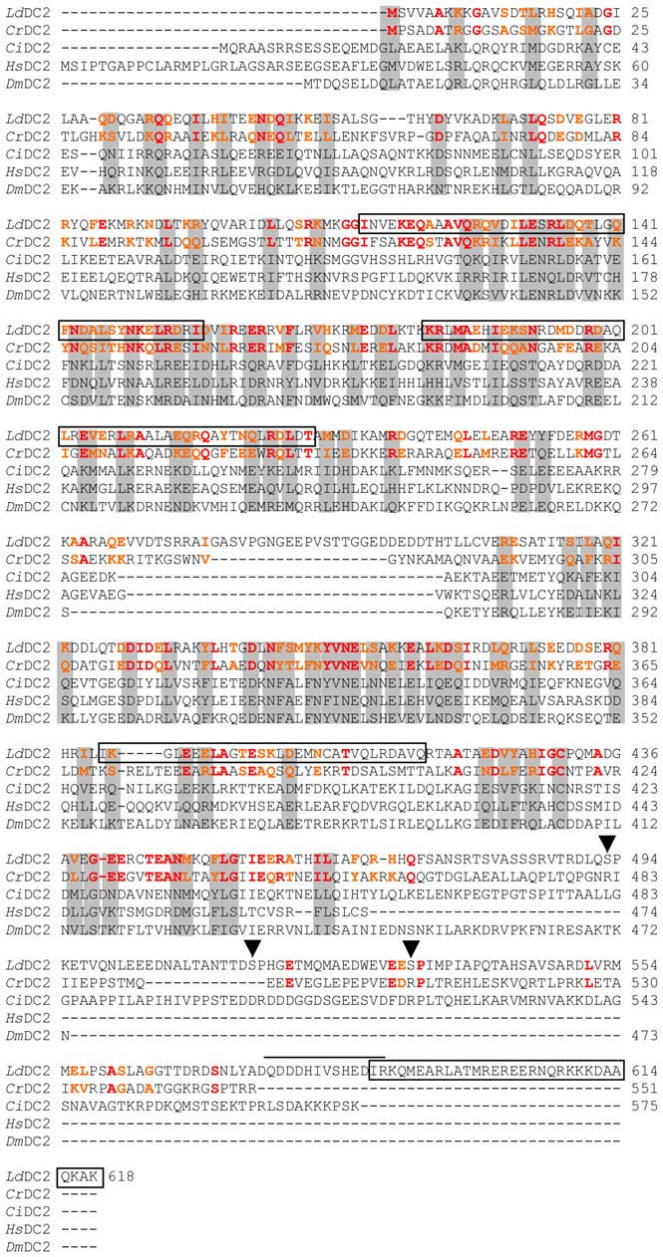
Alignment of DC2 from *L. donovani* with DC2 amino acid sequences from other organisms. *Ld*DC2, *L. donovani* DC2 (GeneBank accession no. GQ240808) *Cr*DC2, *Chlamydomonas reinhardtii* DC2 (GeneBank accession no. AAK72125); *Ci*DC2, *Ciona intestinalis* DC2 (GeneBank accession no. BAB88833); *Hs*DC2, *Homo sapiens* DC2 (GeneBank accession no. AK057357), *Dm*DC2, *Drosophila melanogaster* DC2 (GeneBank accession no. AAF55345). Conserved sequences are marked in grey. Between *Ld*DC2 and *Cr*DC2 identical amino acids are highlighted in red, conserved amino acids in orange. Regions of *Ld*DC2 predicted to form coiled-coil structures are marked with a frame. The EF hand motif is highlighted with a bar. Potential MAP kinase phosphorylation sites are marked with an arrow head.

Figure 1 shows a comparison of the amino acid sequences of the identified *L. donovani* protein with *Cr*DC2 and four additional DC2 proteins from other organisms. *Cr*DC2 has a high α-helical content and comprises three regions with a high probability to form coiled-coil structures [14]. This is a structural motif in which α-helices are coiled together like the strands of a rope creating a so called superhelix. *Cr*DC2 is thought to interact with the other two subunits of the complex *via* this structure. The PAIRCOIL program (http://paircoil.lcs.mit.edu/cgi.bin/paircoil) indicates that the *L. donovani* homologous also has several regions predicted to form coiled-coil structures. These are between amino acids 114–157, 184–228, 386–415 and 586–618 (Fig. 1). In addition, the protein contains a calcium binding EF hand motif between amino acid 576–588 and three potential MAP kinase SP phosphorylation motifs in the C-terminal part of the protein with potential phosphorylation sites at residues S493, S515 and S532.

Because of the demonstrated function of DC2 in *C. reinhardtii*, a flagellar localization of the protein in *L. donovani* was predicted. It was shown earlier that the transport of proteins into the flagella of trypanosomatid organisms is mediated through specific signal sequences [22]–[24]. However, we could not find any of the described motifs within the sequence of the investigated protein.

Supplementary to *Cr*DC2, homologous in other kinetoplastid species such as *Trypanosoma brucei* (45% identity) and *T. cruzi* (42% identity) were found. Additional homologous could be identified in *Micromonas sp.* (23–29% identity), *Ciona intestinalis* (26% identity), *Paramecium tetraurelia* (26% identity), *Tetrahymena sp.* (20–23% identity), *Giardia lamblia* (23% identity), *Drosophila sp.* (22–24% identity) and also in humans (20–22% identity). Figure 1 shows an alignment of the *L. donovani* protein to the *C. reinhardtii*, the *Ciona intestinalis*, one *Drosophila* and one human homologous. All of the proteins are similar in their predicted size and the identities extend throughout the whole sequences with the *L. donovani* protein showing the highest similarity (26%) to *Cr*DC2. No homologous proteins could be found in organisms lacking motile flagella or cilia such as yeast or *Arabidopsis*. In *Caenorhabditis elegans* only a protein that shares 20% homology over the first 300 amino acids can be identified (GeneBank accession no. CAA50183). This molecule, which is termed IF-2 (MUA-6, cytoplasmic intermediate filament), is localized in the hypodermis. It is required for hypodermal integrity and the attachment of muscles to the body wall [25]. So far, there is no hint that a *Cr*DC2 homologous protein exist in *C. elegans*.

Based on these phylogenetic data, we propose that the identified protein is the 70 kDa subunit of the ODA-DC of *L. donovani* and we named it *Ld*DC2 accordingly.

### Expression pattern and intracellular localization *of Ld*DC2

**Figure 2:**
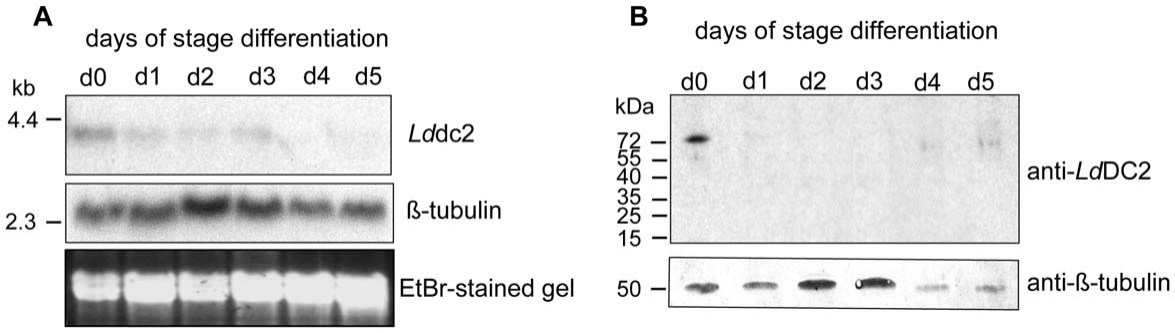
Expression pattern of *Ld*DC2 on RNA and protein level. (**A**) 10 µg mRNA isolated from *in vitro* stage differentiated *L. donovani* day 0 to day 5 was separated in a formaldehyde gel transferred to a nylon membrane and hybridized with a *lddc2* and a *tubulin* probe (loading control). (**B**) Western blot analysis from *in vitro* stage differentiated *L. donovani* day 0 to day 5. 5×10^6^ cells were lyzed directly in hot SDS sample buffer separated on 10% SDS-PAGE transferred to a nitrocellulose membrane and probed with anti-*Ld*DC2 polyclonal antibodies and anti-ß-tubulin monoclonal antibodies (loading control). Molecular standards are indicated on the left.

In order to investigate the expression pattern of *Ld*DC2 Northern blot analysis with RNA isolated from day 0 to day 5 of the *in vitro* stage differentiation of *L. donovani* was performed. The complete coding region of *Ld*DC2 was used as a probe. This analysis revealed a ca. 4 kb transcript which showed a decreasing intensity in the course of differentiation (Fig. 2A).

Protein amounts were examined with the help of a Western blot analysis of cellular extracts from all days of stage differentiation. For this, the full-length *Ld*DC2 protein was synthesized in *E. coli* as an N-terminally His-tagged protein. Matrix-assisted laser desorption ionization time of flight mass spectrometric analysis of the product after digestion with trypsin confirmed the identity of r*Ld*DC2 (data not shown). A polyclonal chicken antiserum generated against r*Ld*DC2 detected a band with an estimated size of about 73 kDa that was exclusive to the promastigote stage (day 0) of the parasite (Fig. 2B).

**Figure 3:**
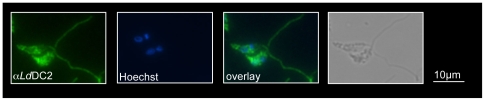
Intracellular localization of *Ld*DC2. *Ld*DC2 was localized by immunofluorescence microscopy. *L. donovani* promastigote parasites were fixed to glass slides and processed for IFA with anti-*Ld*DC2 polyclonal antibodies. DNA was stained with Hoechst. Phase contrast images of the preparations are also included.

The subcellular localization of the protein was determined by indirect immunofluorescence microscopy. The *Ld*DC2 antibodies stained the flagella of promastigote parasites (Fig. 3). We could also detect signals within the cytoplasm of the cells. Since this staining was also observed in amastigotes (data not shown), it may be due to a cross- reactivity of the antibodies that is specific for immunofluorescence.

### Replacement of the *lddc2* gene in *L. donovani* promastigotes

**Figure 4:**
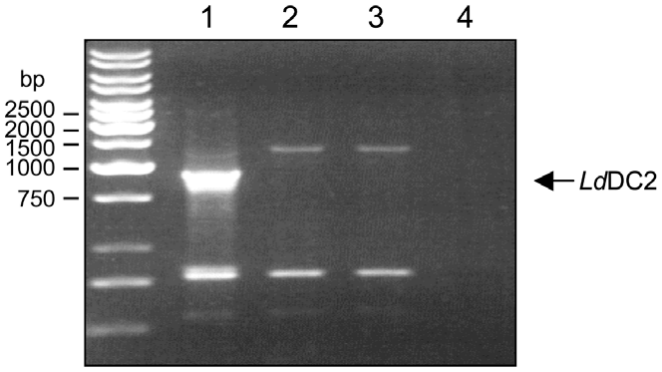
PCR results for Δ*lddc2* ^n/p^ null mutants. 1×10^5^ *L. donovani* promastigotes were incubated for 3 minutes at 95°C in a volume of 20µL. 1µL of the lyzed cells were used for PCR amplification with the specific oligonucleotides CAB-S27 and CAB-AS27 and the obtained DNA fragments were analyzed on a 1% agarosegel. Lane 1, *L. donovani* WT; lane 2, clone Δ*Ld*DC2^n/p^-1; lane 3, clone Δ*Ld*DC2^n/p^-2; lane 4, H_2_O control. Molecular standards are indicated on the left.

**Figure 5:**
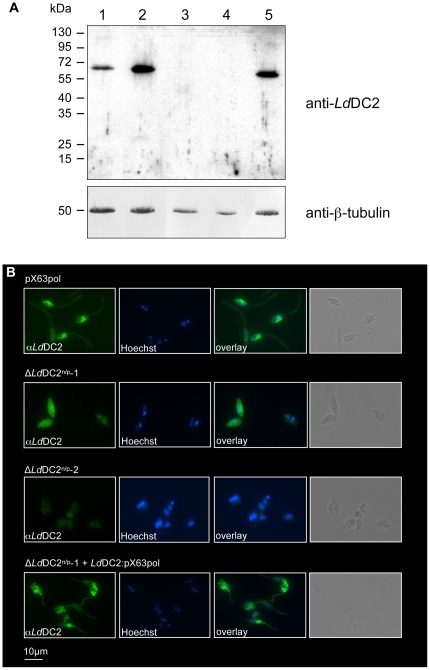
Western blot and immunofluorescence analyzes of Δ*Ld*DC2^n/p^ null mutants. (**A**) 5×10^6^ cells from different *L. donovani* cell lines (Lane 1, *L. donovani* WT; lane 2, *Ld*DC2:pX63pol; lane 3, Δ*Ld*DC2^n/p^-1; lane 4, Δ*Ld*DC2^n/p^-2; lane 5, Δ*Ld*DC2^n/p^-1 + *Ld*DC2:pX63pol) were lyzed directly in hot SDS sample buffer separated on 10% SDS-PAGE transferred to a nitrocellulose membrane and probed with anti-*Ld*DC2 polyclonal antibodies and anti-tubulin monoclonal antibodies (loading control). Molecular standards are indicated on the left. (**B**) *L. donovani* promastigote parasites of the different cell populations (pXpol63; Δ*Ld*DC2^n/p^-1; Δ*Ld*DC2^n/p^-2; Δ*Ld*DC2^n/p^-1 + *Ld*DC2:pX63pol) were fixed to glass slides and processed for IFA with anti-*Ld*DC2 polyclonal antibodies. DNA was stained with Hoechst. Phase contrast images of the preparations are also included.

In order to further characterize the function of *Ld*DC2 in *L. donovani* a null mutant of the gene was generated. Due to the lack of sequence information of the *L. donovani* genome, primers deduced from the untranslated regions of the *L. major dc2*-gene were used to amplify the respective 5′ - and 3′ -UTRs of *L. donovani*. The generated PCR products showed 95% (3′ UTR) and 97% (5′ UTR) identity to the *L. major* sequences. These products were employed to assemble transfection vectors to induce homologues recombination events in *L. donovani*. After successful ligation of the selection markers *neomycinphosphotransferase* and *puromycinacetyltransferase*, the two constructs Δ*lddc2:neo* and Δ*lddc2:pac* were used to transfect *L. donovani* promastigotes. Drug resistant parasites were cloned and the selected cells checked for the presence of *lddc2*. Figure 4 shows the PCR results for two independent Δ*lddc2* ^n/p^ null mutant clones. No specific *lddc2* fragment (1800 bp) could be generated (Fig. 4). The two additional DNA fragments of 750 and 2300 bp are unspecific side products produced by cross reactions of the primers with other regions of the *L. donovani* DNA. Both clones (Δ*lddc2* ^n/p^-1 and Δ*lddc2* ^n/p^-2) were used for further experiments. In order to test whether the successful replacement of both alleles of the *lddc2* gene is accompanied by the loss of the corresponding protein, a Western Blot analysis was performed. The *Ld*DC2 antiserum did not detect the protein in the lysates from the two null mutants Δ*lddc2* ^n/p^-1 and Δ*lddc2* ^n/p^-2 (Fig. 5A). Immunofluorescence assays (IFAs) showed the same results (Fig. 5B). Only parasites transfected with the control plasmid pX63pol showed the typical staining of the flagellum (Fig. 5B) whereas the null mutants did not exhibit any staining of the flagella. They only display an unspecific cytoplasmic staining probably due to cross reactions of the antibodies (Fig. 5B).

A reconstitution of the null mutants through episomal expression of *lddc2* (Δ*Ld*DC2^n/p^-1 + *Ld*DC2:pX63pol) restored the expression of the protein within the cells as shown by Western blot analysis (Fig. 5A) and IFAs (Fig. 5B).

### *Lddc2* null mutants show reduced growth rates and an altered phenotype

**Figure 6:**
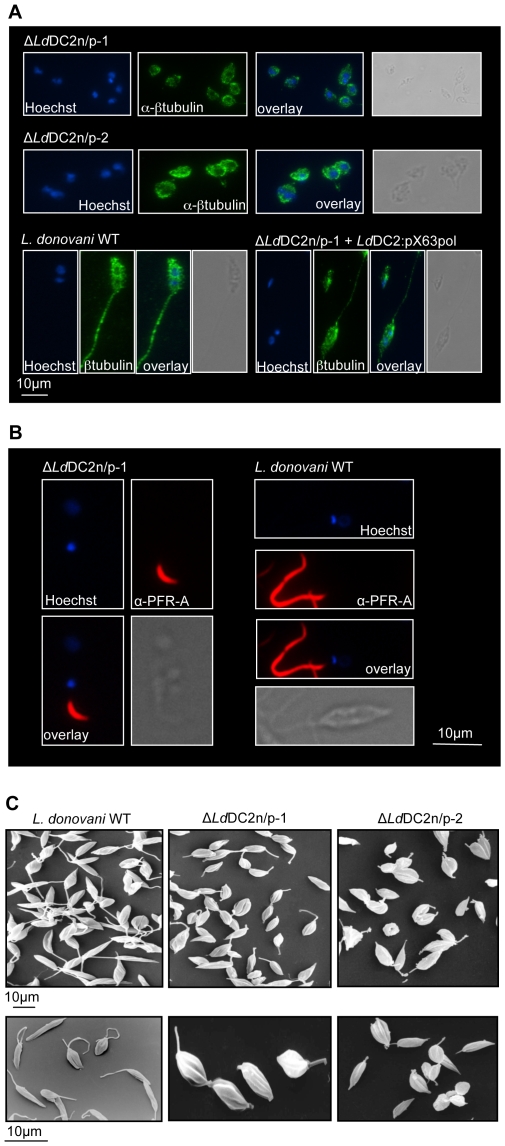
Phenotypical analysis of Δ*Ld*DC2^n/p^ null mutants. (**A**) *L. donovani* promastigote parasites of the different cell populations (Δ*Ld*DC2^n/p^-1; Δ*Ld*DC2^n/p^-2; *L. donovani* WT; Δ*Ld*DC2^n/p^-1 + *Ld*DC2:pX63pol) were fixed to glass slides and processed for IFA with anti-ß-tubulin monoclonal antibodies. DNA was stained with Hoechst. Phase contrast images of the preparations are also included. (**B**) The different *L. donovani* cell populations (Δ*Ld*DC2^n/p^-1; WT) were fixed to glass slides and processed for IFA with anti-PFR2 monoclonal antibodies. DNA was stained with Hoechst. Phase contrast images of the preparations are also included. (**C**) The different *L. donovani* cell populations (WT; Δ*Ld*DC2^n/p^-1; Δ*Ld*DC2^n/p^-2) were analyzed with scanning electron microscopy. The lower panel represents magnifications of the different cells.

**Figure 7:**
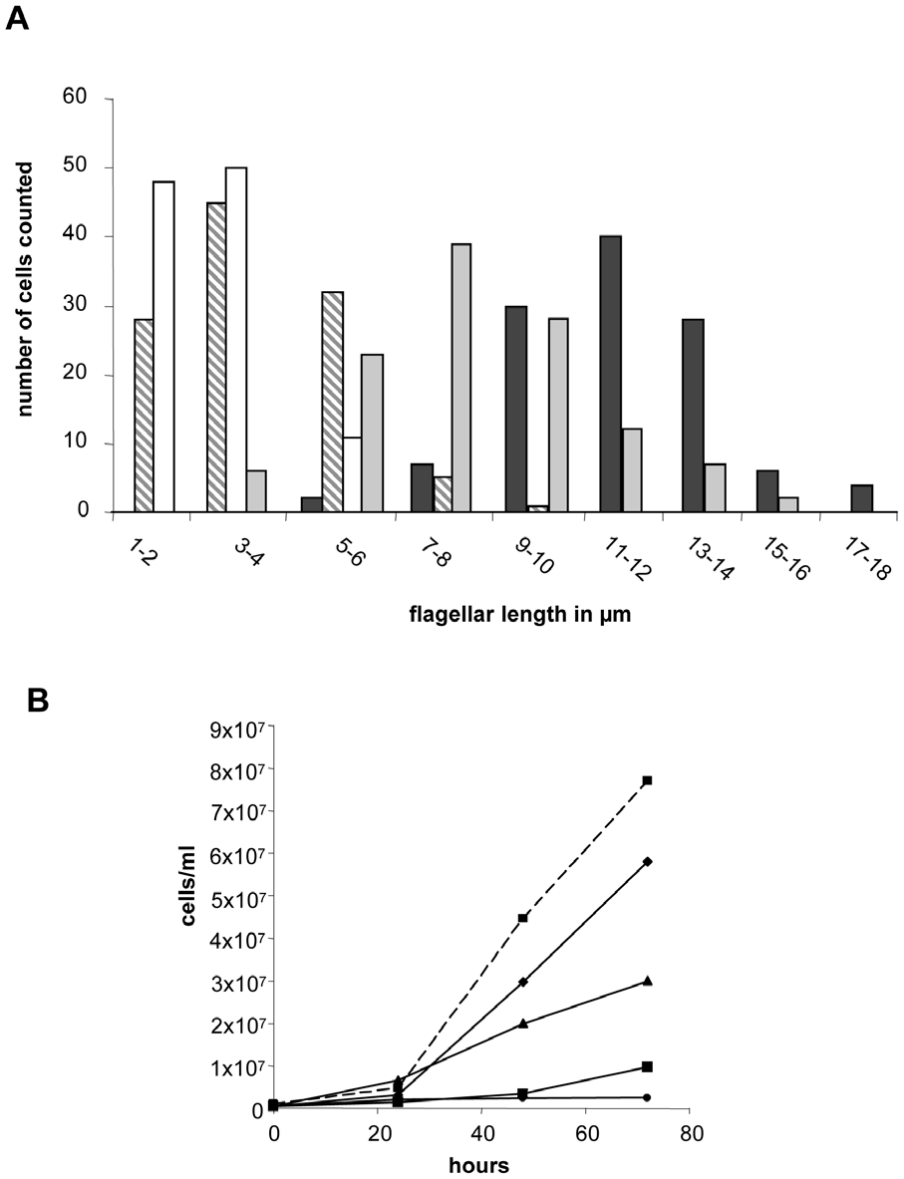
Flagellar length and growth rates of Δ*Ld*DC2^n/p^ null mutants. (**A**) Histograms of flagellar length from Δ*Ld*DC2^n/p^ null mutants and WT *L. donovani* parasites (black bars, *L. donovani* WT; grey bars, Δ*Ld*DC2^n/p^-1; white bars, Δ*Ld*DC2^n/p^-2; striped bars, Δ*Ld*DC2^n/p^-1 + *Ld*DC2:pX63pol). (**B**) Growth rates of Δ*Ld*DC2^n/p^ null mutants. The different *L. donovani* cell populations (WT (square with dashed line); Δ*Ld*DC2^+/n^ (triangle); Δ*Ld*DC2^n/p^-1 (circle); Δ*Ld*DC2^n/p^-2 (square with solid line); Δ*Ld*DC2^n/p^-1 + *Ld*DC2:pX63pol (diamond)) were cultured for 4 days and cells were counted every 24 hours with a Casy Cell Counter (Schärfe System).

A striking consequence of the *lddc2* gene replacement is an altered cell shape and flagellar length. To document the aberrant morphology, IFAs with a β-tubulin specific antibody were performed. The microscopic images of this analysis are shown in Figure 6A. The two mutants Δ*lddc2* ^n/p^-1 and Δ*lddc2* ^n/p^-2 exhibit a rounded cell shape and a drastically reduced flagellum. By contrast, WT and reconstituted mutants show the normal promastigote spindle shaped form with long flagella (Fig. 6A). The average mean flagellar length of WT promastigotes (n = 119) was 11.6±2.4 µm whereas the mutant parasite lines displayed a mean flagellar length of only 3.7±1.4 µm (Δ*lddc2* ^n/p^-1, n = 137) and 2.9±1.0 µm (Δ*lddc2* ^n/p^-2, n = 140). Transgenic expression of *lddc2* (Δ*lddc2* ^n/p^-1+*Ld*DC2:pX63pol, n = 119) restores flagellar length (9.5±3.4 µm, Fig. 7A). In addition, we performed IFAs with an antibody directed against the flagellar protein PFR2 (Fig. 6B). Once more, the reduced flagellar length in the null mutants can be clearly observed. Scanning electron microscopic analysis of WT parasites and null mutants confirmed the observed phenotype (Fig. 6C).

**Figure 8:**
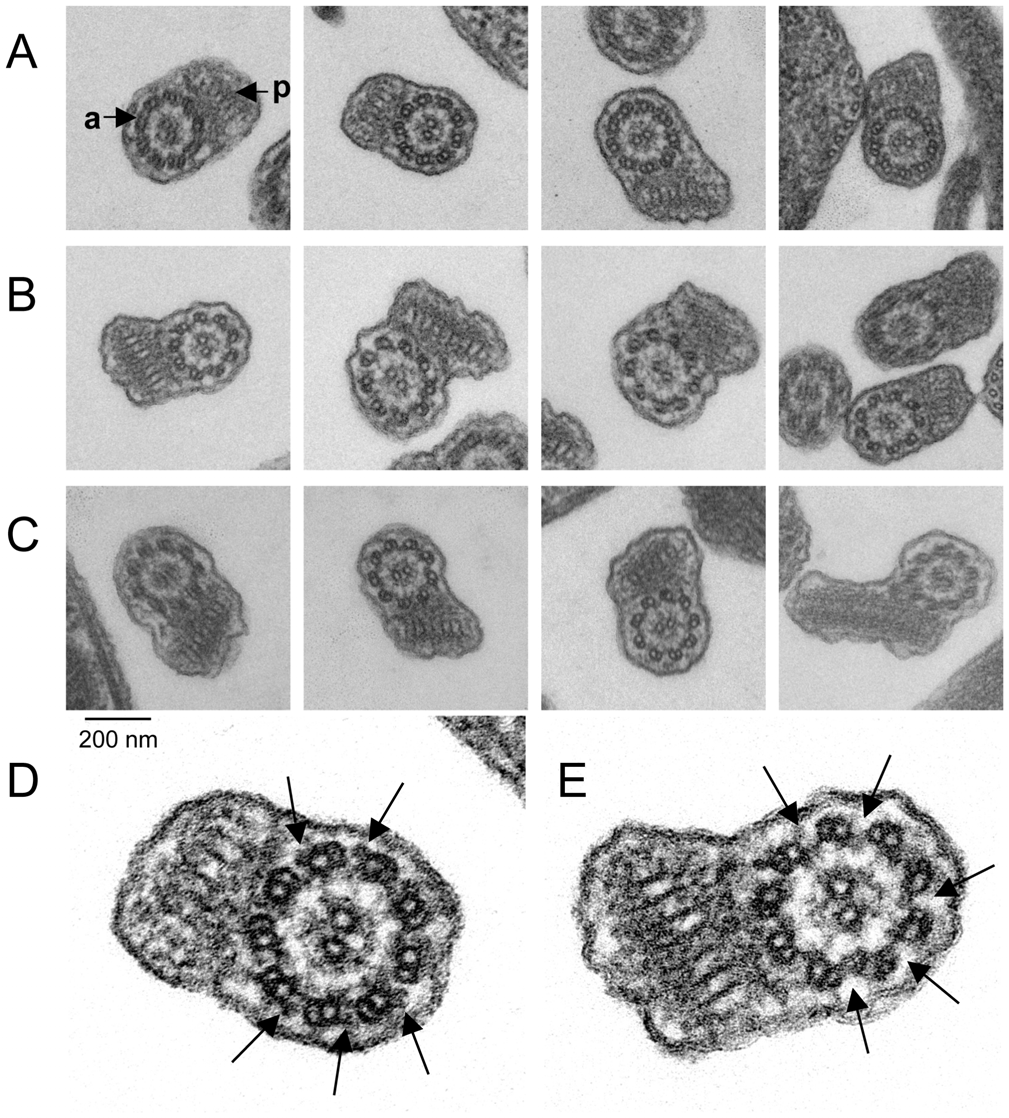
Ultrastructure of Δ*Ld*DC2^n/p^ null mutants flagella. Electron microscopic studies of cross-sections from flagella of chemically fixed *L. donovani* WT (A), Δ*Ld*DC2^n/p^-1 (B) and Δ*Ld*DC2^n/p^-2 (C) promastigotes at the same magnification. Magnifications of *L. donovani* WT (D) and Δ*Ld*DC2^n/p^-1 (E). Outer dynein arms (arrows) are missing in the mutant. a, axoneme; p, paraflagellar rod.

Using transmission electron microscopy on cross-sections of chemically fixed promastigote cells, the flagellar ultrastructure was examined. Interestingly, the flagellar ultrastructure of the null mutants was changed. As shown in Figure 8, the outer dynein arm is present in all WT cells analysed. However, it is missing in the two mutants Δ*lddc2* ^n/p^-1 and Δ*lddc2* ^n/p^-2. The absence was observed in all analyzed flagellar cross section (15 per cell line) apart from two sections of mutant Δ*lddc2* ^n/p^-1.

A striking difference between WT cells and mutants was observed concerning the motility. While the wild-type *L. donovani* promastigotes show directed movement across the microscopic field of vision, the mutants, while wiggling in place, are unable to translocate for any distance (Video S1, S2, S3, supporting information).

Another distinctive feature of *Lddc2* null mutants was a strongly reduced cellular growth (Fig. 7B). Doubling times for the null mutants were ∼80 h, roughly eight times longer than those of WT or of the reconstituted null mutant (8–12 h doubling time). A population of single-allele gene replacement mutants (Δ*lddc2* ^+/n^) showed an intermediary phenotype with a doubling time of approximately 20 h.

**Figure 9:**
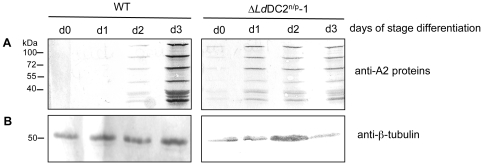
Expression of amastigote-specific proteins in Δ*Ld*DC2^n/p^ null mutants. Western blot analysis from *in vitro* stage differentiated *L. donovani* WT and Δ*Ld*DC2^n/p^ mutants from day 0 to day 3. Cells were lyzed directly in hot SDS sample buffer separated on 10% SDS-PAGE transferred to a nitrocellulose membrane and probed with anti-A2 monoclonal antibodies (**A**) and anti-tubulin monoclonal antibodies (loading control) (**B**). Molecular standards are indicated on the left.

As both null mutant clones display more resemblance to amastigote than promastigote parasites regarding their morphological shape and growth rates, we looked for the expression of known amastigote marker proteins. Wild type and Δ*lddc2* ^n/p^-1 parasites were subjected to stage conversion conditions for three days. Lysates from these *in vitro* differentiated cells were tested for the presence of the A2-protein family. Expression of the A2-gene family is a hallmark of the *L. donovani* amastigote stage [26] and is commonly used as marker for amastigote differentiation. Figure 9 shows a Western blot analysis using anti-A2 monoclonal antibodies. Due to the very different growth rates cell densities for the null mutants were lower. Nevertheless, expression of the A2 gene family can be detected even from day 0 in the null mutants, while wild type parasites do not show detectable A2 protein before day 2. Thus, null mutants express trace levels of A2 protein in the promastigote which increase rapidly by day 1 and do not change until day 3 (Fig. 9A).

### *Lddc2* null mutants show slightly increased *in vitro* infectivity

**Figure 10:**
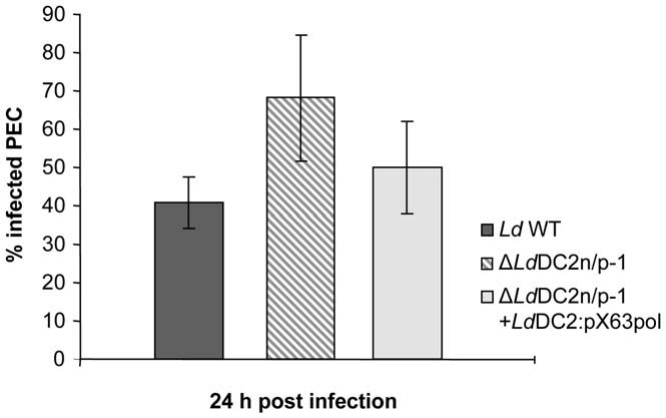
PEC infection assay of Δ*Ld*DC2^n/p^ null mutants. Adherent mouse peritoneal exsudate cells were incubated with *L. donovani* WT, Δ*Ld*DC2^n/p^-1 and Δ*Ld*DC2^n/p^-1 + *Ld*DC2:pX63pol parasites at a ratio of 1∶10. After 24 hours cells were fixed, stained with Giemsa and analyzed microscopically.

PEC infection assays were used to analyze the involvement of *Ld*DC2 in infectivity of *L. donovani*. WT, Δ*lddc2* ^n/p^-1 and the reconstituted mutant parasites were incubated with mouse peritoneal exudate cells (PEC) for 24 hours and examined for intracellular amastigote load. Figure 10 shows the results of three independent experiments. The percentage of infected PECs for the WT parasites is 41±0.07% on average. The examined null mutant line caused an average percentage of 68.13±0.16%. The reconstituted mutant showed an average percentage of 50±0.12% infected PECs. The null mutant therefore revealed an increased infection. It is slightly higher than the one of WT parasites. The infectiousness of reconstituted mutant parasites was reduced again. At 24 h, the majority of WT parasites had not been phagocytized yet and were still seen as extracellular promastigotes attached to the host cells. Those cells were not counted. Reconstituted mutants showed a similar phenotype. By contrast, Δ*Ld*DC2 mutants were detected mostly as intracellular amastigotes.

## Discussion

In the course of a proteome analysis of the *in vitro* stage differentiation of *L. donovani* a subunit of the outer dynein arm docking complex (ODA-DC) was identified as amastigote-specific [18]. A Western blot analysis with an antibody raised against the respective recombinant protein however showed the protein exclusively in the promastigote stage of the parasite. Due to the suspected function of the protein, an amastigote-specific expression can not be anticipated because the parasite only exhibits a rudimentary flagellum during this life cycle stage. Our previous data showed that the theoretically expected and the experimentally determined molecular weights of the identified protein differ greatly. The calculated molecular mass of *Ld*DC2 is 70,000. The protein detected in the 2D-gels of the proteome analysis displayed a molecular weight of ∼35 kDa only [18]. It is possible that the protein detected was a degradation product of *Ld*DC2 accumulated during degeneration of the flagellum in the course of differentiation. However, one would expect to detect this degradation product in the performed Western blot analysis of the stage differentiation (Fig. 2B). This is not the case. We suspected that the recognized epitopes are not functional within the degradation product formed during amastigote differentiation.

The proteome analyses of the *in vitro* stage differentiation of *L. mexicana* identified the paraflagellar rod protein 2c as amastigote-specific [27]. Here, too, only a fragment of the protein was detected. Apparently, protein degradation products formed during the differentiation into amastigotes can be detected for at least five days.

Northern blot analysis of the *lddc2* expression showed a decreasing intensity of the transcript during stage conversion with signals no longer detectable at days 4 and 5. The Western Blots, by contrast, showed intact *Ld*DC2 protein only in the promastigotes. This indicates that either *Ld*DC2 mRNA is no longer translated or that degradation is upregulated once stage conversion commences.

Immunofluorescence studies displayed a flagellar localization of the protein, confirming the predicted function of *Ld*DC2. Additional staining could be detected in the cytoplasm of the parasites. It is not clear whether this is due to a pool of non-assembled material or if it is an unspecific side reaction of the antiserum.

The ODA-DC subunit identified in this study is the 70 kDa subunit DC2. The protein sequence showed altogether four regions with a high probability to form coiled-coil structures. The function of these structures is usually related to the formation of homo- and heterodimers [28]. For the homologous protein from *C. reinhardtii* it was shown that these regions are responsible for the interaction of *Cr*DC2 with another subunit of the ODA-DC, DC1 [14]. DC1 contains similar structural motifs and is associated with DC2 [13].

The C-terminal part of *Cr*DC2 contains a short glutamic acid rich repeat followed by a region with a high content of charged amino acids. It was postulated that the interaction with the tubulins of the outer dynein arms as well as with the intermediate chain take place *via* this region. *Ld*DC2 contains a similar region, albeit shorter than in the *C. reinhardtii* homologous. In contrast to *Cr*DC2 an additional EF hand motif close to the C-terminus could be identified for the *L. donovani* protein. This motif is also present in other trypanosomatid DC2 proteins as for example in *L. braziliensis*, *T. brucei* and *T. cruzi*. However, the function of this motif is unclear. The third subunit of the complex DC3, also contains such sequence motifs. It was proposed that the protein is involved in the Ca^2+^ dependent regulation of the activity of the outer dynein arm [12].

While searching the *L. major* protein database homologous for all subunits of the ODA-DC could be found supporting the concept that the outer dynein arms in *Leishmania* are also anchored to the A-tubule *via* an ODA-DC. The composition of flagella and cilia show a remarkable conservation throughout the evolution [29].

A large number of proteins are needed for the correct assembly of a flagellum. A proteomic analysis of purified *C. reinhardtii* flagella identified 360 proteins with high confidence and another 292 with moderate confidence [7]. Broadhead and colleagues investigated the flagellar proteome of *T. brucei* and found it to be constituted of at least 331 proteins [30]. All these flagellar components must to be imported from the cytoplasm as flagella do not contain their own ribosomes. It was shown that specific signal sequences mediate the transport of proteins into the flagella of kinetoplastid organism [22]–[24]. However, dynein arms are assembled within the cytoplasm prior to transport [31], restricting the need for signal sequences to a few proteins within those large complexes. No known flagellar import signal sequence could be identified within the *Ld*DC2 sequence, indicating that it is transported together with other components of the ODA-DC.

The *lddc2* null mutants showed a variety of morphological changes as well as a reduced growth rate. Parasites lacking *Ld*DC2 were considerably smaller, with a rounded cell shape. The flagella were shortened, and parasites were not as motile as wildtype promastigote *L. donovani*. The mutants are unable to translocate for any distance. Instead they wiggle around in one place. The *oda1* mutant of *C. reinhardtii* showed a similar flagellar phenotype [14],[32],[33]. These cells lack the outer dynein arm and the ODA-DC. They were isolated initially because of their slow swimming phenotype with a reduced frequency and force of their flagellar beating after a chemical mutagenesis [32]. Later on it was shown that this phenotype was due to a mutation within the *crdc2* gene leading to the generation of a stop-codon right after the translation initiation site [14]. The lack of *Ld*DC2 in *Leishmania* causes a much stronger phenotype. The null mutation not only affects the motility of the cells, but their entire morphology including flagellar length and ultrastructure. Indirect immunofluorescence microscopy, light microscopy, and scanning electron microscopy all confirm that the *lddc2* null mutant displays reduced flagellar length. To analyze this phenotype more closely transmission electron microscopy of flagellar cross-sections was performed. The flagellar ultrastructure shows that like in the *oda1* mutant of *C. reinhardtii* the flagella lack the outer dynein arm. Apart from this the mutants possess a normal axoneme and the typical PFR structure. Immunofluorescence studies also confirmed the presence of the paraflagellar rod protein PFR2 in the null mutants.

Several studies have shown that the reduction of flagellar correlated with a change in overall cell morphology in other trypanosomatid organisms. The disruption of the cytoplasmic dynein-2 heavy chain gene *DHC2.2* in *L. mexicana* resulted in immotile parasites with a rounded cell body. Ultrastructural analysis revealed short flagella that lacked the paraflagellar rod and contained a disorganized axoneme [6]. In *Chlamydomonas* and *C. elegans*, cytoplasmic dynein-2 is one of the motor proteins that power the intraflagellar transport (IFT) [34], a bidirectional active transport of components required for the flagellar assembly. *LmxDHC2.2* seems to be required for maintenance of promastigote cell shape and correct assembly of the flagellum. A similar phenotype could be observed in RNAi generated knock-down mutants of IFT proteins in trypanosomes [35]. Down-regulation of IFT leads to assembly of a shorter flagellum. Cells with a shorter flagellum are smaller, with a direct correlation between flagellum length and cell size. The deletion of the ADF/cofilin gene in *Leishmania* likewise results in non-motile cells with reduced flagellar length and severely impaired beat frequency. The PFR is not assembled, vesicle-like structures appear throughout the flagellum and actin distribution is altered markedly [36]. It was speculated that ADF/cofilin driven actin dynamic activity is required for intracellular trafficking of flagellar proteins from the cytoplasm to the flagellar base. Deletion mutants of the MAP kinase homologue MPK3 in *L. mexicana* also leads to reduced flagellar length, stumpy cell bodies and vesicle and membrane fragments in the flagellar pocket [37]. The authors speculate that *Lmx*MPK3 might be involved in the regulation of IFT. The absence of a correct PFR structure in all described mutants suggests that the IFT is severely impaired and this might be responsible for the observed phenotypes as PFR assembly seems to be mediated by IFT [22],[38]. *Ld*DC2 null mutants do not lack the PFR. Therefore, the observed reduction of the flagellum and the abnormal cell morphology seems to be a consequence of another mechanism.

In 2003, Wiese *et. al.* postulated that a MAP kinase kinase of *L. mexicana* (*Lmx*MKK) is involved in the regulation of flagellar length in promastigote cells. The gene is promastigote-specific and a null mutant showed shortened flagella. The mutants were able to induce lesions during an infection of BALB/c mice, albeit with delay [39]. In addition, as already described, null mutants of the MAP kinase 3 of *L. mexicana* (*Lmx*MPK3) also possess shortened flagella. Contrary to the MKK knock-out MPK3 is not required to establish an infection in mice [37]. It is not known so far how flagellar length is regulated. Since over 80 phosphorylated proteins were identified in the flagella of *Chlamydomonas* [40],[41] the involvement of protein kinases and classical signal transduction pathways is quite likely. The amino acid sequence of *Ld*DC2 contains three potential MAP kinase phosphorylation sites in the C-terminal region, and the homologous protein in *L. mexicana* (having the same phosphorylation sites) is indeed phosphorylated. However, *in vitro* kinase assays using *in vivo* activated *Lmx*MPK3 and *Ld*DC2 showed that the ODA-DC subunit most likely is not a substrate for MPK3 (Erdmann, personal communication). Additional phosphorylation studies will be necessary to clarify the regulatory mechanisms underlying flagellar length control.

Another consequence of *Ld*DC2 knock-out was the deregulated expression of the amastigote-specific protein family A2. Expression can already be detected in the promastigote cells with an increase early during differentiation. If and by which mechanism(s) the loss of a structural protein of the flagellum influences the expression of other proteins remains to be clarified. The degeneration of the flagellum however is a central event during differentiation into the amastigote stage, and it is conceivable that the accumulation of other flagellar proteins in parasites that cannot assemble full-length flagella may cause unfolded protein stress and thus mimic the heat stress that is the key signal for stage conversion [42].

The infectivity of *Ld*DC2 null mutants was slightly increased compared to wild type *L. donovani*. We could show that at 24 h after infection, most wild type parasites were attached to the outside of the host cells and only a limited percentage of the host cells showed intracellular parasites. For the *Ld*DC2 null mutants we saw higher rates of infection and fewer extracellular parasites could be found. The interaction between *Leishmania* and their host cells is very complex. The two major surface molecules involved in macrophage binding are GP63, a surface metallo protease and various phosphoglycans including LPG (Lipophosphoglycan) [43],[44]. LPG molecules form a dense glycocalyx on the surface of the promastigotes, including the flagellum. Both molecules, GP63 and LPGs, are virulence factors essential for the survival of *L. major* in the insect vector as well as in the vertebrate host [45]–[47]. Zhang and Matlashewski showed that the A2 proteins constitute bona fide virulence factors. Antisense-mediated reduction of A2 protein synthesis in *L.* donovani caused a greatly reduced infectiousness *in vitro* and *in vivo* [26],[48]. Furthermore, expression of A2 proteins in *L.* major which lacks these genes changed the pathology of *L.* major [48]. Therefore, the increased expression of the A2 protein family in the *Ld*DC2 null mutants may account for the increased infection rates.

An equivalent gene replacement in *L. major* should allow the use of a mouse infection model to test whether the changes observed *in vitro* with *L. donovani* are reflected in the animal host.

In summary, we can conclude that the correct assembly of the flagellum has a great influence on the investigated characteristics of *Leishmania* parasites. The lack of only one flagellar protein leads to a completely different morphology and slows down proliferation. In addition, the parasite's ability to invade host cells is slightly enhanced. It will be interesting to see whether the lack of other structural proteins of the flagella may have a similar impact.

## Supporting Information

Video S1 Wild-type promastigotes.
(2.35 MB MOV)

Video S2 Δlddc2^n/p^-1.
(2.17 MB MOV)

Video S3 Δlddc2^n/p^-2.
(2.64 MB MOV)
