# Expression of Concern: Characterization of a Subunit of the Outer Dynein Arm Docking Complex Necessary for Correct Flagellar Assembly in *Leishmania donovani*

**DOI:** 10.1371/journal.pntd.0010981

**Published:** 2022-12-09

**Authors:** The PLOS Neglected Tropical Diseases Editors

After this article [1] was published, concerns were raised about similarities between some lanes and vertical discontinuities in the western blot images in Figures 2 and 5. Specifically:

- Areas of the background appear highly similar in lanes d1, d2 and d3 in the anti-LdDC2 panel in Figure 2B; double bands appear to be present in lanes d2 and d3 in the underlying image available via a link in a Publisher’s Note posted on the article on 25th September, 2017, but do not appear to be present in the published figure panel.
- In both panels in Figure 5a there appear to be vertical discontinuities between lanes 1 and 2, and between lanes 2 and 3.

During editorial follow-up, the last author supported the conclusion reported in [1] that the “polyclonal chicken antiserum generated against rLdDC2 detected a band with an estimated size of about 73 kDa that was exclusive to the promastigote stage (day 0) of the parasite”, and stated that the light bands in lanes d2 and d3 of the underlying blot for Figure 2B (S1 File) are known non-specific cross-reactions that frequently appear at that mass even in the absence of the target antigen. Replicate blots for Figure 2B from the time of the original experiments are provided here in S2 and S3 Files.

The authors also provided the following information about the production and validation of the antibodies:

First, the pre-immune serum is checked for possible cross-reactivities or non-specificities. If the pre-immune serum is clean, the corresponding animal is immunized according to the scheme given in article [1] and the antibodies are purified. The antibodies are tested by western blot against the recombinant protein. If this is not yet detected, further booster immunization follows. Subsequently, the serum is checked again for recognition of the putative target in corresponding extracts (from *Leishmania*). A titration to optimize recognition by the antibody is performed. For the immunofluorescence tests, additional controls are performed with only the first or second antibody. The immune serum reacts with the recombinant protein (CAB).

An Academic Editor reviewed the data and advised that the presence of a signal in lanes containing amastigote cells lacking flagella in the underlying blot for Figure 2B (S1 File) raises a concern about the support for the conclusion that this protein is only found in the promastigote stage of the parasite expressing a flagellum, but also noted it is possible that the additional bands are due to a non-specific interaction with another protein. The consulting Academic Editor further noted that the immunohistochemical staining of the flagella of promastigotes with the same antisera in Figure 3 of [1] appears to show staining predominantly in the cell body rather than the flagellum, which does not fully support the conclusion that this is a promastigote and flagellum-specific protein.

Regarding the specificity of the antiserum, the consulting Academic Editor advised that although it is shown that in the western blots, the band appears close to the predicted size of the native LdDC2 protein, the antibody validation methodology described does not fully address the specificity of the antisera, noting that it is possible the antisera binds to the native LdDC2 protein and also binds non-specifically to other native *Leishmania* proteins. There do not appear to be blots showing antisera staining of recombinant protein in article [1], so it is not possible to assess how the staining of the recombinant protein compares to the native protein. However, the consulting Academic Editor also noted that the data reported in Figure 5 show that the western blot signal and the immunohistochemical staining disappear in the null mutants, providing compelling support that the antisera are specific. The Academic Editor advised that there are some inconsistencies in the results shown in the article [1], but based on Figure 5 it appears that the antisera are specific.

During editorial follow-up, the authors provided the original underlying data for both panels in Figure 5a (S4 and S5 Files). The last author stated that lane 2 was removed in both blots because it contained a sample that was not important for the experiment. S5 File shows that lane 4 was also removed in both blots. The concerns around the vertical discontinuities in Figure 5a are therefore resolved.

The authors also provided the original underlying data for both panels in Figure 9a (S6 File). The original underlying data for Figure 9B is no longer available. The remaining data are available from the last author upon request.

In light of the issues with Figure 2B compared to the underlying blot, the *PLOS Neglected Tropical Diseases* Editors issue this Expression of Concern due to concerns about the integrity of data reporting in this article.

## Supporting information

S1 File Figure 2B anti-LdDC2 panel. (JPG)

S2 File Figure 2B replicate 1. (JPG)

S3 File Figure 2B replicate 2. (JPG)

S4 File Figure 5A anti-LdDC2 panel. (JPG)

S5 File Figure 5A anti-b-tubulin panel. (JPG)

S6 File Figure 9A both panels. (JPG)
