# Supplementary figures and images for: Expression of Concern: Characterization of a Subunit of the Outer Dynein Arm Docking Complex Necessary for Correct Flagellar Assembly in Leishmania donovani
Source: PLoS Negl Trop Dis. 2022 Dec 9;16(12):e0010981. doi: 10.1371/journal.pntd.0010981 (PMC9733864; doi:10.1371/journal.pntd.0010981)

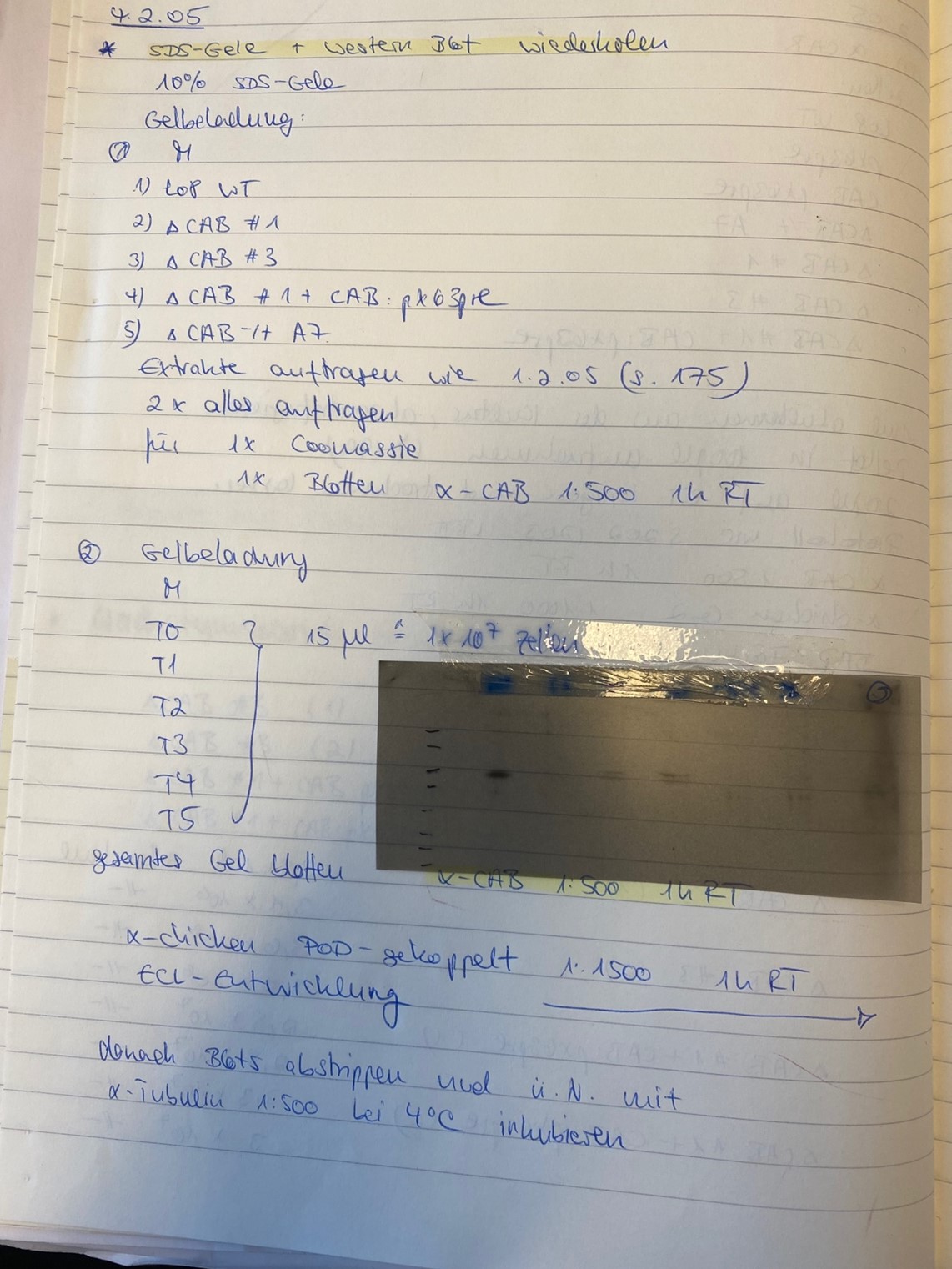

Supplement: S1 File — (JPG) [file pntd.0010981.s001.jpg]

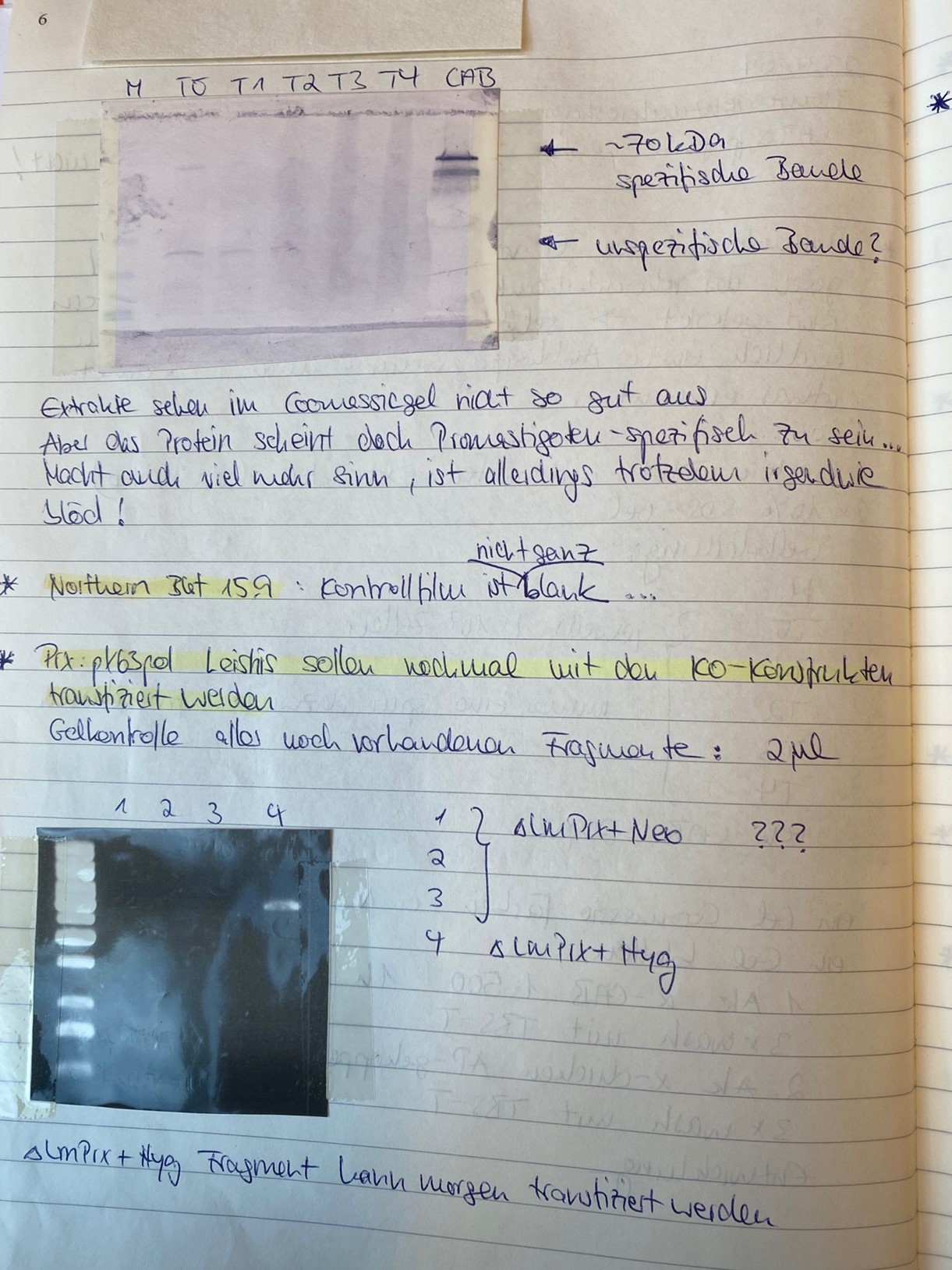

Supplement: S2 File — (JPG) [file pntd.0010981.s002.jpg]

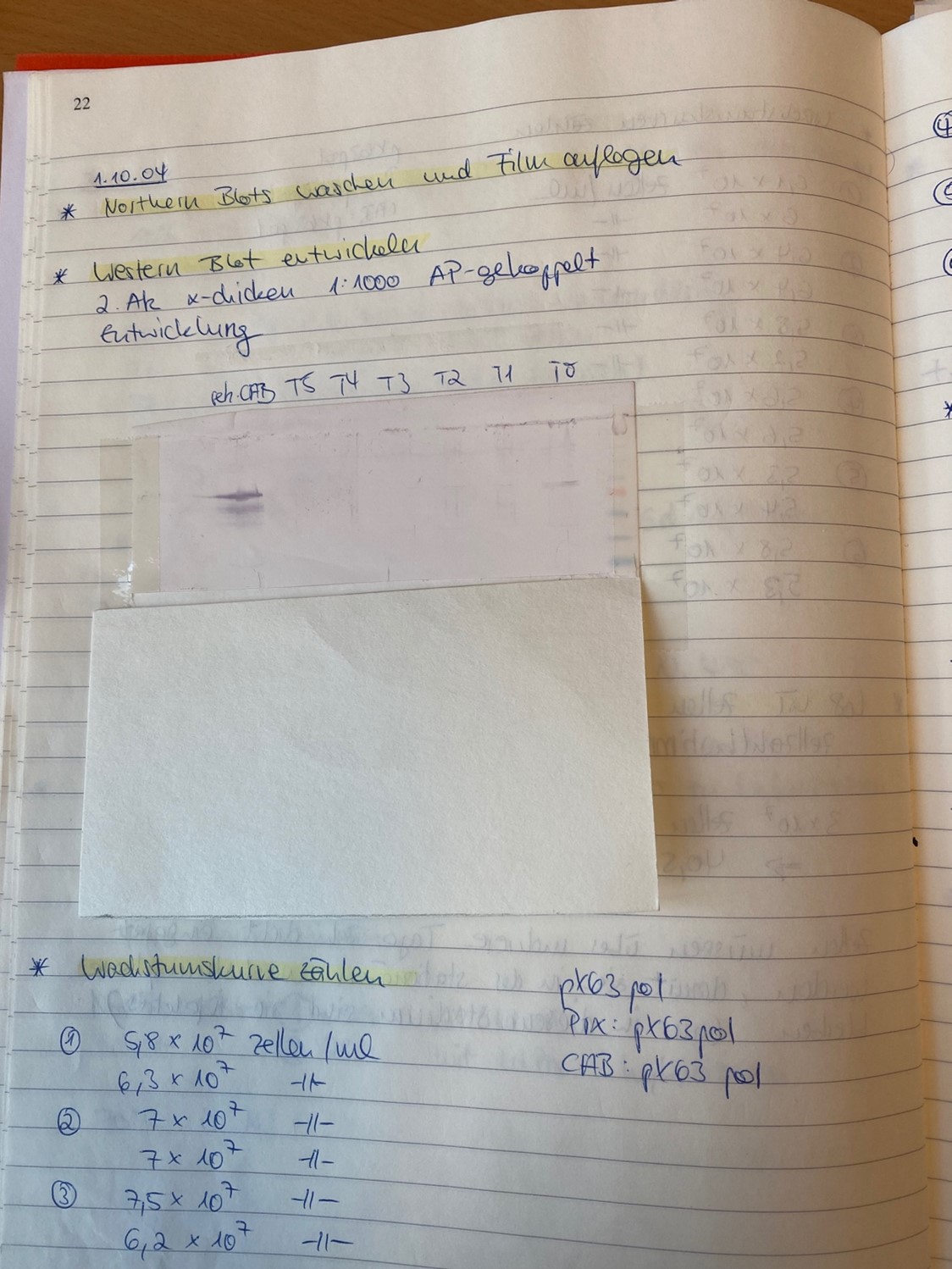

Supplement: S3 File — (JPG) [file pntd.0010981.s003.jpg]

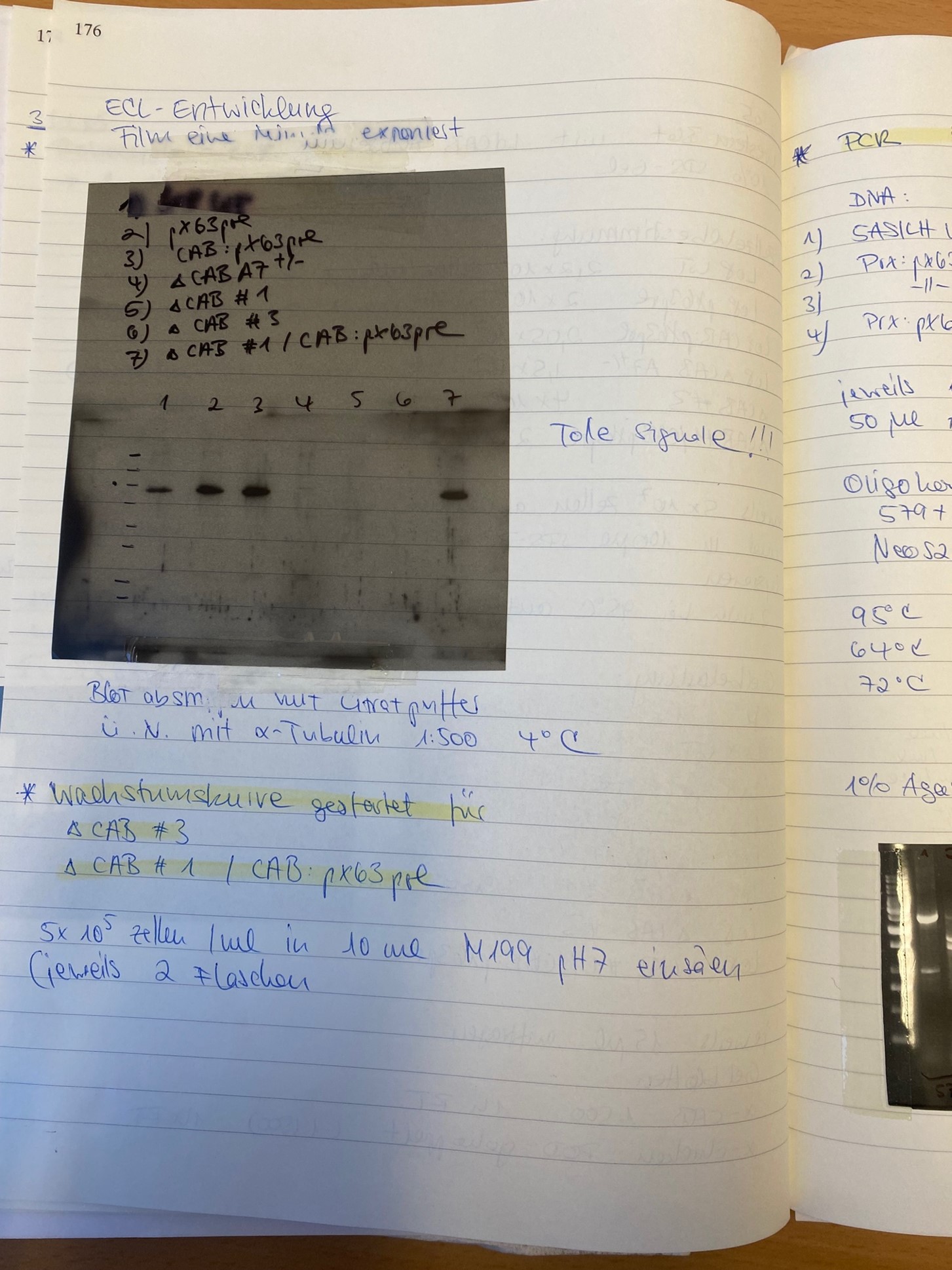

Supplement: S4 File — (JPG) [file pntd.0010981.s004.jpg]

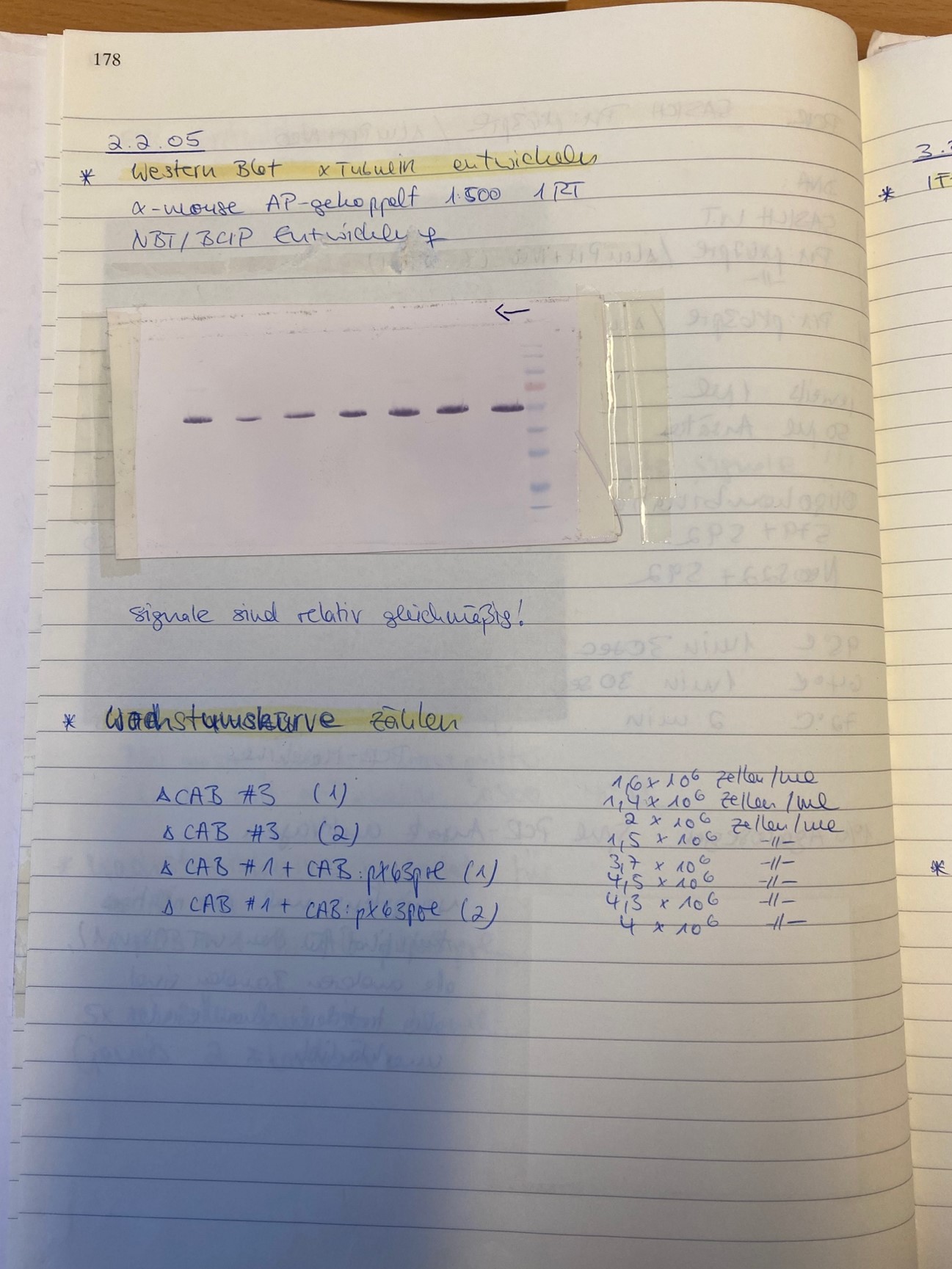

Supplement: S5 File — (JPG) [file pntd.0010981.s005.jpg]

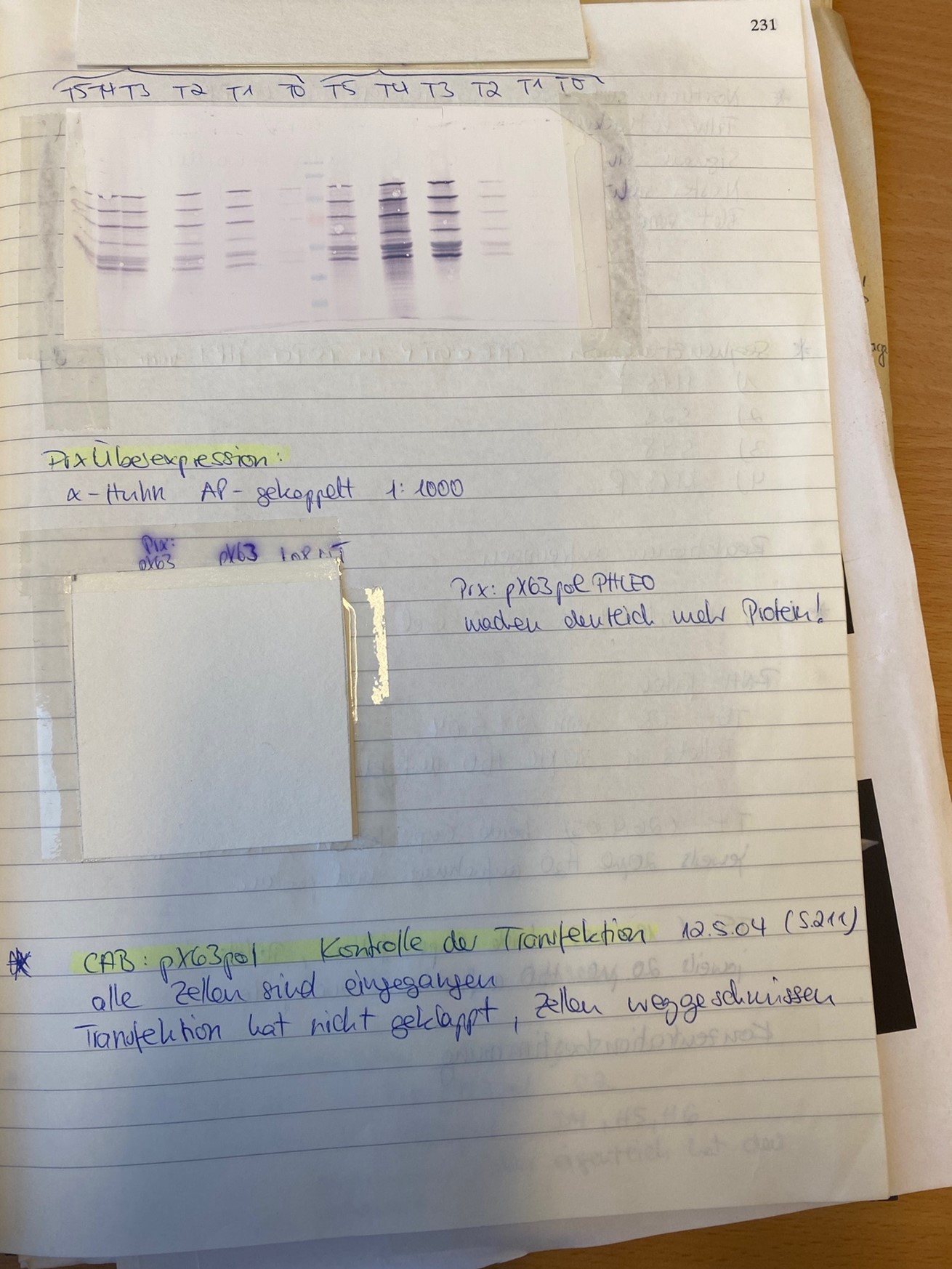

Supplement: S6 File — (JPG) [file pntd.0010981.s006.jpg]
